# Measuring health-related quality of life in cervical cancer patients: a systematic review of the most used questionnaires and their validity

**DOI:** 10.1186/s12874-016-0289-x

**Published:** 2017-01-26

**Authors:** Casper Tax, Marlie E. Steenbergen, Petra L. M. Zusterzeel, Ruud L. M. Bekkers, Maroeska M. Rovers

**Affiliations:** 1 Department of Operating Rooms, Radboud University Medical Centre, Radboudumc Institute for Health Sciences, P.O. Box 9101, 6500 HB Nijmegen, The Netherlands; 2 Radboud University Faculty of Medicine, Nijmegen, The Netherlands; 3 Department of Gynaecology, Radboud University Medical Centre, Radboudumc Institute for Health Sciences, P.O. Box 9101, 6500 HB Nijmegen, The Netherlands; 4 Department of Health Evidence, Radboud University Medical Centre, Radboudumc Institute for Health Sciences, P.O. Box 9101, 6500 HB Nijmegen, The Netherlands

**Keywords:** HRQoL, Cervical cancer, Psychometric properties, Validity

## Abstract

**Background:**

Data on health-related quality of life (HRQoL) is paramount for shared and evidence based decision-making. Since an overview of cervical cancer HRQoL tools and their validity appears to be lacking, we performed a systematic review on usage of disease specific HRQoL instruments in cervical cancer patients and their psychometric properties to identify the most suitable cervical cancer specific HRQoL tool.

**Methods:**

We searched Pubmed, EMBASE and PsycINFO from inception up to 18 October 2016 for studies on quality of life in cervical cancer patients. Data extraction and HRQoL identification was performed by two independent reviewers. Validation studies of the identified cervical cancer specific HRQoL tools were retrieved and assessed on psychometric properties using the COSMIN checklist. All used cervical cancer specific HRQoL instruments were scored and ranked according to their psychometric properties.

**Results:**

We included 156 studies (20,690 patients) and identified 31 HRQoL tools. The EORTC QLQ-CX24 (35 studies; 5,556 patients) and FACT-Cx (22 studies; 4,224 patients) were the only cervical cancer specific tools.

The EORTC QLQ-CX24 had 4 out of 9 positive rated psychometric properties; internal consistency, content and construct validity, and agreement. Criterion validity, reliability, and interpretability scored doubtful. Responsiveness and floor- and ceiling effects were not reported. The FACT-Cx had 2 out of 9 positive rated psychometric properties; internal consistency and agreement. Content validity, reliability, and interpretability scored doubtful while criterion and construct validity scored negative. Responsiveness and floor- and ceiling effects were not reported.

**Conclusion:**

The validity of the often used EORTC QLQ-CX24 questionnaire for cervical cancer patients remains uncertain as 5 out of 9 psychometric properties were doubtful or not reported in current literature. Cervical cancer specific HRQoL tools should therefore always be used in conjunction with validated generic cancer HRQoL tools until proper validity has been proven, or a more valid tool has been developed.

**Electronic supplementary material:**

The online version of this article (doi:10.1186/s12874-016-0289-x) contains supplementary material, which is available to authorized users.

## Background

Treatment of the fourth most common cancer in women, cervical cancer, consists of either surgery and/or (chemo-)radiotherapy, based on the FIGO stage of the disease [1, 2]. Depending on the treatment, different side effects, such as: bladder, bowel, and vaginal dysfunction, lymphedema, and lymphocysts can occur [3, 4]. These side effects, together with the emotional and social impact of the disease, influence a patients’ health-related quality of life (HRQoL), even when survival is extended. The acceptance of medical treatments is critically dependent on these HRQoL consequences, making it one of the most important parameters in the evaluation of medical treatments.

Quality of life is a complex, multidimensional construct, with a range of conceptual definitions and is often evaluated using HRQoL tools. There is general agreement that multidimensional HRQoL assessment should at least include physical, social and psychological/emotional functioning and well-being [5]. The validity and suitability of such HRQoL tools, is represented by their psychometric properties. Psychometric properties indicate if a measurement tool is; free of error (reliability), assesses what it is intended to measure (validity), is able to detect change in an individual over time (responsiveness), and the degree to which one can assign qualitative meaning to quantitative scores (interpretability) [6]. Using an instrument with (good) psychometric properties that have been evaluated enables the user to draw more robust and substantial conclusions. Since the psychometric properties of a measurement tool can differ per target population, it is recommended that they are evaluated in that specific target population.

Others have studied the psychometrics and appropriateness of HRQoL tools in gynaecologic oncology in general [7–9]. However, both a clear overview of the available disease specific HRQoL tools for cervical cancer patients and an appraisal of their psychometric properties are lacking. Vistad et al [10] reported on the impact of cervical cancer on HRQoL, and critically appraised a number of studies regarding HRQoL measurement in this population. However, they did not report or evaluate the psychometric properties of the HRQoL instruments for cervical cancer. Furthermore, FIGO stage is important in the treatment of cervical cancer, and can result in different side effects influencing relevant aspects of HRQoL [2–4, 11]. As such, the validity, reliability, and responsiveness of an HRQoL tool can differ per disease stage. For a valid and patient-centered evaluation of health status, it is important that the HRQoL tool measures aspects of health status that are important to patients with cervical cancer, and that the measurement characteristics are adequate for the specific patient population. Thus, we hypothesized that cervical cancer-specific HRQoL tools will provide the most valid and patient-centered evaluation of health status.

The aim of this study therefore is to provide an overview of the used HRQoL tools in cervical cancer patients, to identify cervical cancer specific HRQoL tools and to assess their psychometric properties. This allows for an evidence based choice of a cervical cancer specific HRQoL tool in both clinical practice and clinical trials.

## Methods

### Data source and search

We systematically searched EMBASE, Pubmed, and PsycINFO from inception up to 18 October 2016 for studies on quality of life assessment in cervical cancer patients. The search strategy combined synonyms for cervical cancer, questionnaires and quality of life, see Additional file 1 for the complete search strategy. All citations were imported into the bibliographic database of EndNote X5 (Thomas Reuters, New York, NY, USA).

### Study selection

After retrieving all the records in Endnote, duplicates were removed and records were screened on title and abstract for relevance by two independent reviewers (M.E.S and C.T.). Inclusion criteria for full text assessment were; (1) assessing quality of life in (2) patients with cervical cancer using (3) an HRQoL tool [5], and (4) availability of a full text (5) peer reviewed article. For example, studies focusing on a single quality of life domain were excluded as the concept of HRQoL is multidimensional. Whenever a full text questionnaire was not available, the corresponding authors were contacted for a copy in order to assess if the questionnaire did meet the HRQoL definition. In case of disagreement, a third reviewer was consulted (M.M.R.). All studies that were included from the systematic review were documented as supplemental references and contain the prefix ‘s’, followed by the respective reference number.

### Data extraction, synthesis and analysis

Two independent reviewers (M.E.S. and C.T.) extracted the following data: HRQoL tool, number of cervical cancer patients, and their respective FIGO stage (Additional file 2). An overview was made of all used HRQoL tools with the number of included patients. A distinction was made between HRQoL tools for the following domains: generic HRQoL, HRQoL for cancer in general, cervical cancer-specific HRQoL, other cancer-specific HRQoL, and other non-cancer but disease or symptom specific HRQoL tools. Depending on the data presentation, we defined the following stages as early stage cervical cancer; stage I, OR stage IA, IB and IIA, OR stage IA1 + 2, IB1 and IIA1.

When two or more HRQoL tools were used in one study, each HRQoL tool was included either in combination or as separate tool, based on their use. Thus, the reported number of studies and/or patients can exceed the total overall included number of studies and/or patients from the systematic search. In case of disagreement, a third reviewer was consulted (M.M.R).

### Psychometric property assessment

As we hypothesized that cervical cancer-specific HRQoL tools will provide the most valid and patient-centered evaluation of health status, we only assessed psychometric properties of the identified cervical cancer-specific HRQoL tools. Psychometric property assessment of the cervical cancer specific HRQoL tools was based on all available studies in which one or more psychometric properties of the tool were assessed and reported for cervical cancer patients. These studies were identified through the references of studies that were already included after the first search for HRQoL tools used in cervical cancer patients and by searching the official website of the specific HRQoL tool. Furthermore, we also searched Embase, Pubmed, and PsycINFO using a search strategy that combined synonyms and terms for cervical cancer, validation studies/psychometrics and quality of life (Additional file 3). We also performed a reference and related article search.

The psychometric properties were assessed according to the COSMIN (COnsensus-based Standards for the selection of health Measurement Instruments) criteria published by Terwee et al. [12] including content validity, internal consistency, criterion validity, construct validity, reproducibility (agreement, reliability), responsiveness, floor- and ceiling effects, and interpretability. A scoring model was used based on a positive (+), doubtful (?), or negative (-) rating that was given to each psychometric property [13]. If more than one validation study assessed the same psychometric property, the best rating was used as recommended by the COSMIN protocol. Unfortunately, there are no methods available to pool results on psychometric property testing from different validation studies while taking their underlying methodological quality (weight) into account. We therefore reported all ratings in order to provide a clear overview of the best rating and the variation between validation studies for each psychometric property. If no information was found on the psychometrics, it was not assessable and was scored with an “X”. See Additional file 4 for the definition of the psychometric properties and their scoring criteria.

The ratings were not used for a total sum score per HRQoL tool as each individual psychometric property can have its own weight regarding the quality and the suitability of the cervical cancer specific HRQoL tools [12].

## Results

**Fig. 1:**
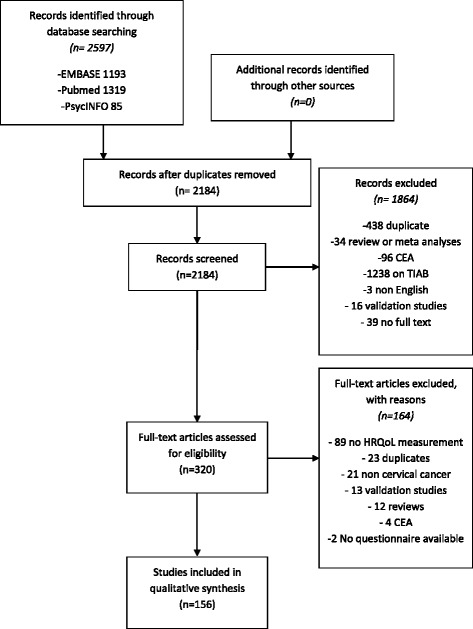
Flow diagram of the study selection

Figure 1 provides an overview of the literature search and study selection. Our literature search yielded 2184 unique records, of which 320 remained after screening titles and abstracts. The full-text of these studies was reviewed for eligibility. Studies were excluded for the following reasons: not including an HRQoL assessment (89), duplicates (23), non-cervical cancer patients (21), validation study (13), review (12), a cost-effectiveness study (4), and no full text copy of the questionnaire available (2). This yielded 156 studies (20,690 patients) using 31 different HRQoL tools. See Additional file 5 with the supplemental references for a list of all included studies and their respective reference number, with the prefix ‘s’.

### HRQoL tools, type and frequency

The 31 identified HRQoL tools were categorized as generic (*n* = 10), cancer-specific (*n* = 9), and cervical cancer-specific-tools (*n* = 2). Other disease specific HRQoL tools that were used in cervical cancer patients were related to ovarian (*n* = 1), bladder (*n* = 1), breast (*n* = 1), or brain cancer (*n* = 1), or to chronic illness (*n* = 2), terminal care (*n* = 1), rectal bleeding (*n* = 1), fecal incontinence (*n* = 1), or menopause (*n* = 1). Table 1 provides an overview of the identified tools, their abbreviations, their frequency, and number of patients assessed with that tool.

**Table 1:** HRQoL tools, number of times used in literature, full name and identified validation studies

	Studies	Early stage	Advanced stage	Overall	Full name
General					
SF-36	21	1025	436	3452	36-Item Short Form health survey [s1–s21]
WHOQOL-BREF	9	415	281	963	WHO Quality of Life – Abbreviated [s22–s30]
SF-12	8	569	443	1263	12-Item Short Form health survey [s31–s38]
EQ-5D	7	655	363	2287	Euroqol-5D [s21, s39–s44]
VR 12	1	0	0	1016	Veterans Rand 12-Item Health Survey [s20]
SF8	1	311	218	529	8-Item Short Form health survey [s43]
PROMIS	4	294	108	565	Patient Reported Outcomes Measurement Information System [s45–s48]
QL-I	1	34	45	79	Quality of Life Index [s49]
PGWB	1	24	22	46	Psychological General Well-Being [s50]
ALLTAG	1	24	22	46	[German] Questionnaire on everyday life [s50]
Total	54	3351	1938	10246	
Cancer					
FACT-G	26	634	2126	3807	Functional Assessment of Cancer Therapy – General [s15, s33, s51–s74]
EORTC QLQ-C30	29	344	561	2792	European Organization for Research and Treatment of Cancer, Quality of Life Questionnaire, Core module [s13, s14, s49, s75–s97]
FACT NTX	3	0	1108	1108	Functional Assessment of Cancer Therapy/Gynecologic Oncology Group-Neurotoxicity [s69, s70, s72]
FACT-GP	2	147	54	221	Functional Assessment of Cancer Therapy - General Population [s47, s48]
QOL-CS	2	0	0	72	Quality of Life – Cancer Survivors [s17, s100]
CARES-SF	1	0	0	129	Cancer Rehabilitation Evaluation System – Short Form [s93]
FACT AN	1	0	115	115	Functional Assessment of Cancer Therapy-Anemia [s73]
SDCQ	1	0	0	59	Socio-demographic and Clinical Questionnaire [s101]
MQOLS-CA	1	0	0	38	Multidimensional Quality of Life. Scaled Cancer [s102]
Total	66	1125	3964	8341	
Cx specific					
EORTC QLQ-CX24	35	3212	1508	5556	European Organization for Research and Treatment of Cancer, Quality of Life Questionnaire, Cervical cancer module [s18, s21, s103–s135]
FACT-Cx	22	1231	2973	4224	Functional Assessment of Cancer Therapy – Cervical cancer [s32, s38, s46–s48 s69–s72, s136–s148]
Total	57	4443	4481	9780	
Other cancer specific					
Ovarium specific					
ORTC QLQ-OV28	1	0	0	27	European Organization for Research and Treatment of Cancer, Quality of Life Questionnaire, Ovarian cancer module [s96]
Bladder Cancer					
FACT BL	1	0	0	13	Functional Assessment of Cancer Therapy – Bladder cancer [s149]
Breast Cancer					
FACT-B	1	0	0	10	Functional Assessment of Cancer Therapy – Breast cancer [s150]
Brain Cancer					
FACT-BR	1	0	1	1	Functional Assessment of Cancer Therapy – Brain cancer [s151]
Total	4	0	1	51	
Other					
Terminal care					
MVQOLI	1	0	32	32	Missoula-VITAS Quality of Life Index [s152]
Menopause					
WHQ 30	1	0	0	15	Woman’s Health Questionnaire [s153]
Chronic illness					
FACT-SP	2	19	91	114	Functional Assessment of Chronic Illness Therapy - Spiritual Well-Being [s133, s154]
FACT F	1	0	1	1	Functional Assessment of Chronic Illness Therapy-Fatigue [s97]
Rectal bleeding					
RBQOLS	1	0	0	2	Rectal Bleeding Quality of Life Scale [s155]
Fecal incontinence					
FIQLI	1	0	0	21	Fecal Incontinence Quality of Life [s156]
Total	7	19	124	185	
Total Overall ^a^	156	6848	7053	20690	

^a^ Total numbers differ as a study and/or study population can be counted more than once in and between categories while only counting once for the overall total

The two cervical cancer specific HRQoL tools used were the EORTC QLQ-CX24 and FACT-Cx, which were used in 35 and 22 studies including 5,556 and 4,224 patients, respectively. In 3 studies (*n* = 574 patients) the EORTC QLQ-CX24 was combined with another, mostly a more generic HRQoL tool (Table 2). The FACT-Cx was combined with another HRQoL tool in 8 studies (*n* = 1,915 patients). The EORTC QLQ-CX24 was used in 68% early FIGO stage cervical cancer patients as compared to 29% with the FACT-Cx,

**Table 2:** Cervical cancer specific HRQoL tool usage

	Studies	Early stage	Advanced stage	Overall
As single tool				
EORTC QLQ-CX24	32	2836	1350	4982
FACT-Cx	14	706	1603	2309
EORTC QLQ-Cx24 in combination with ^a^				
EQ-5D & SF-36	1	247	39	291
SF-36	1	110	28	173
FACT Sp	1	19	91	110
FACT-Cx in combination with ^b^				
NTX	3	0	1108	1108
PROMIS	3	294	108	422
SF-12	2	231	154	385

^a^ The QLQ-C30 core questionnaire did not count as a combination

^b^ The FACT-G core questionnaire did not count as a combination

### Psychometric properties

Table 3 shows the psychometric properties of the EORTC QLQ-CX24 and FACT-Cx. For the EORTC QLQ-CX24 and FACT-Cx, 7 and 3 validation studies were available with study populations ranging from 100 to 860 patients.

**Table 3:** Psychometric properties of the cervical cancer-specific HRQoL tools

FACT-CX								Overall
Study	Ding [21]	Fernandes [22]	Fregnani [23]					
Country	China	Brazil	Brazil					
Number of participants	400	149	100					
Content validity	?	X	?					?
Internal consistency	+	+	+					+
Criterion validity	?	X	-					-
Construct validity	-	X	-					-
Agreement	X	X	+					+
Reliability	?	X	?					?
Responsiveness	X	X	X					X
Floor- & ceiling effects	X	X	X					X
Interpretability	?	X	?					?
EORTC QLQ-CX24								Overall
Study	Greimel [14]	Jayaskera [17]	Singer [19]	Hua [16]	Shin [18]	Du Toit [15]	Paradowska [20]	
Country	multiple	Sri Lanka	Germany	China	Korea	South Africa	Poland	
Number of participants	167	112	134	115	860	208	171	
Content validity	+	?	?	?	X	?	?	+
Internal consistency	+	+	+	+	+	+	+	+
Criterion validity	?	X	?	?	?	?	?	?
Construct validity	X	+	X	X	X	X	X	+
Agreement	X	X	X	X	X	X	+	+
Reliability	?	?	?	?	?	?	?	?
Responsiveness	X	X	X	X	X	X	X	X
Floor- & ceiling effects	X	X	X	X	X	X	X	X
Interpretability	?	?	?	?	?	?	?	?

?; doubtful design/methods thus no quantitative or qualitative scoring possible

X; not reported therefore no quantitative or qualitative scoring possible

### EORTC QLQ-CX24

There is positive evidence regarding the content validity, construct validity, internal consistency, and agreement since the items in the questionnaire were extensively selected involving both patients and investigators; the scores of the tool were related to the treatment status as hypothesized prior to the study; Cronbach’s α was above 0.70; and the test-retest showed an ICC between 0.85 and 0.89, respectively. The criterion validity and reliability are uncertain as a questionable reference standard was used to validate the tool’s score to a reference standard and/or inappropriate statistical methods were used. These methods included: calculating a Cohen’s D, Kruskall Wallis, Mann-Whitney, Wilcoxon, students’ t-test, or ANOVA with a p-value between the subgroups to prove that patients could be distinguished from each other in subgroups, such as early and advanced FIGO stage or treatment status, based on the tool’s score. The interpretability of the EORTC QLQ-CX24 score is limited as a minimal important change was not defined. Responsiveness and floor- and ceiling effects were also not assessed in any of the validation studies. The scoring model resulted in 4 positive, 3 doubtful, and 2 not assessable psychometrics for the EORTC QLQ-CX24, out of a maximum of 9.

### FACT-Cx

There is positive evidence regarding the internal consistency and agreement as items per (sub)scale were correlated with a Cronbach’s α above 0.70 and the test-retest showed an ICC between 0.68 and 0.84. However, there is no evidence available regarding the content validity, i.e. it is unclear how the questionnaire was built and how the specific questions were selected. The reliability of the FACT-Cx is uncertain as again, inappropriate statistical methods were used to prove that patients could be distinguished from each other in subgroups such as early and advanced FIGO stage or treatment status, based on the tool’s score. The interpretability of the FACT-Cx score is limited as a minimal important change was not defined. Responsiveness and floor- and ceiling effects were also not assessed in any of the validation studies. The criterion and construct validity were limited as the correlation α with the studied reference standard (SF-36) was below 0.70 and less than 75% of the hypotheses on how the scores of the questionnaire would relate to other measures in a manner that was consistent with theoretically derived hypotheses were confirmed.

The scoring model resulted in 2 positive, 3 doubtful, 2 not assessable, and 2 negative psychometrics for the FACT-Cx, out of a maximum of 9.

All validation studies included early and advanced stage cervical cancer patients and patients in these subgroups could be distinguished, based on their overall scores. However, the psychometric properties per subgroup were not reported and thus not assessable for neither the EORCT QLQ-CX24 nor the FACT-Cx.

## Discussion

The FACT-Cx and EORTC QLQ-CX24 were the identified cervical cancer-specific HRQoL tools, which were used in 22 and 35 out of 156 studies, respectively. The EORTC QLQ-CX24 appears to be the most used and a more appropriate tool to assess HRQoL in cervical cancer patients. However, its validity is uncertain since 5 out of 9 psychometric properties are doubtful or not reported in current literature. For example, no correlation was found between the performance of the tool and a reference standard, the minimal important change that should be detected was not defined, and floor and ceiling effects were not reported. The validity of the FACT- Cx is even more uncertain as 7 out of the 9 psychometric properties were doubtful or not reported at all. Similar problems as with the EORTC were reported; there was no correlation found between the performance of the tool and a reference standard, the minimal important change that should be detected was not defined, and floor and ceiling effects were not reported. But for the FACT-Cx there was also no description on how the questionnaire and its items were selected, hypotheses regarding the scores were not confirmed, and it remained unclear if repeated measurements over a longer period of time can detect a (relevant) change in quality of life.

Thus the EORTC QLQ-CX24 has been more thoroughly assessed regarding its psychometric properties and scored better (both regarding the number of positively rated psychometric properties and the score per psychometric property) when compared to the FACT-Cx.

To our knowledge, this is the first systematic review that identified different HRQoL tools that have been used to assess quality of life in patients with cervical cancer. We used the COSMIN checklist for a thorough evaluation of the quality of the two cervical cancer specific HRQoL tools. We have provided evidence that the EORTC QLQ-CX24 is the most appropriate and valid cervical cancer-specific HRQoL for a patient-centered evaluation of health status of cervical cancer patients in general.

Our study has a few limitations. First, one possible HRQoL tool, the SES-QOL, could not be included as there was no full text copy of the tool available. Despite repeated requests, we did not receive a full text copy and had to exclude this tool from our results as we could not assess whether it meets the HRQoL definition. On the other hand, as the SES-QOL is not a cervical cancer specific HRQoL tool it would not have influenced our final conclusion.

Second, almost all psychometric properties highly depend on methods and design of the validation study. For instance, a property such as construct validity could score positively if only one hypothesis was tested and confirmed (>75% confirmed), while rated negatively when another hypotheses was tested but rejected (<75% confirmed). Thus the rating of an HRQoL tools’ validity could therefore also be a representation of the validation study design, instead of the actual validity. Regardless, the uncertainty surrounding the validity of cervical cancer-specific HRQoL tools remains, and more evidence is needed to reduce this uncertainty.

Do note that the scoring model that we applied cannot be used to calculate an ‘overall’ score as the weight of each individual psychometric property can differ per specific design, application and/or study population, e.g. construct validity, reliability for discriminating between different (sub)groups, and responsiveness for the evaluation of treatment effects [12]. Thus, an external comparison on validity across HRQoL tools is only possible per psychometric property and not on an ‘overall’ score.

Third, the psychometric properties in the identified studies were often not reported, incorrect or unclear, and the terminology and definitions differed from those proposed by Terwee et al [12]. Regardless, this is the most up-to-date overview of current available literature and it should be noted that the absence of evidence on the above mentioned psychometric properties, either due to no reported data or inappropriate design/methods, is not to be confused with evidence of their absence.

Based on our results, the use of only a cervical cancer-specific HRQoL tool is not preferred since its validity remains to be proven. We therefore recommend to always use a well-validated generic HRQoL tool to be able to ascertain the most valid and patient-centered evaluation of health status, both in clinical practice and clinical trials. In addition, by using already well-validated generic HRQoL tools in combination with one of the cervical cancer-specific tools, researchers will be able to properly assess the psychometric properties of the FACT-Cx and EORTC QLQ-CX24. Another option could be to develop a new and more valid HRQoL tool. However, this may be redundant as there remains uncertainty regarding the validity of already available HRQoL tools and the absence of evidence on their validity should not to be confused with evidence of absent valid tools. For both the validation of current HRQoL tools and development of a new tool, we would recommend to use an established protocol, such as the quality criteria presented by Terwee et al [12]. A data presentation with an assessment of psychometric properties for both early and advanced stage cervical cancer is warranted as their treatment and subsequent possible side effects differ distinctively [14–21].

## Conclusion

The validity of the often used EORTC QLQ-CX24 questionnaire for cervical cancer patients remains uncertain since 5 out of 9 psychometric properties were doubtful or not reported in current literature. Cervical cancer specific HRQoL tools should therefore always be used in conjunction with validated generic cancer HRQoL tools until proper validity has been proven, or a more valid tool has been developed.

## Additional files

Additional file 1: Search strategy systematic review represents the search strategy with combined synonyms for cervical cancer, questionnaires and quality of life in Pubmed, EMBASE and PsycINFO from inception to October 18th 2016. (DOCX 16 kb)
Additional file 2: Data extraction form shows the format of the data extraction form used to systematically collect data per study on HRQoL tool, number of cervical cancer patients, and their respective FIGO stage. (DOCX 12 kb)
Additional file 3: Search strategy validation studies represents the search strategy for validation studies with combined synonyms and terms for cervical cancer, validation studies/psychometrics and quality of life in Pubmed, EMBASE and PsycINFO from inception to October 18th 2016. (DOCX 12 kb)
Additional file 4: Adapted scoring model and scoring criteria shows the definition of the psychometric properties and the adapted scoring criteria from the COSMIN consortium which we applied to rate each cervical cancer specific HRQoL tool. (DOCX 16 kb)
Additional file 5: Supplemental references s1–s146 contains references to all individual studies included in the systematic review. (DOCX 37 kb)

## Abbreviations

COSMIN: COnsensus-based Standards for the selection of health Measurement Instruments
HRQoL: Health-related quality of life
ICC: Intraclass Correlation Coefficient

## References

[1] Ferlay J, Soerjomataram I, Dikshit R, Eser S, Mathers C, Rebelo M, Parkin DM, Forman D, Bray F (2015). Cancer incidence and mortality worldwide: sources, methods and major patterns in GLOBOCAN 2012. International journal of cancer.

[2] Colombo N, Carinelli S, Colombo A, Marini C, Rollo D, Sessa C (2012). Cervical cancer: ESMO Clinical Practice Guidelines for diagnosis, treatment and follow-up. Annals of oncology : official journal of the European Society for Medical Oncology / ESMO.

[3] Achouri A, Huchon C, Bats AS, Bensaid C, Nos C, Lecuru F (2013). Complications of lymphadenectomy for gynecologic cancer. European journal of surgical oncology : the journal of the European Society of Surgical Oncology and the British Association of Surgical Oncology.

[4] Maher EJ, Denton A (2008). Survivorship, late effects and cancer of the cervix. Clinical oncology (Royal College of Radiologists (Great Britain)).

[5] Agency EM: Reflection paper on the regulatory guidance for the use of health-related quality of life (HRQL) measures in the evaluation of medicinal products. 2005.

[6] Mokkink LB, Terwee CB, Patrick DL, Alonso J, Stratford PW, Knol DL, Bouter LM, de Vet HC (2010). The COSMIN study reached international consensus on taxonomy, terminology, and definitions of measurement properties for health-related patient-reported outcomes. Journal of clinical epidemiology.

[7] Preston NJ, Wilson N, Wood NJ, Brine J, Ferreira J, Brearley SG (2015). Patient-reported outcome measures for use in gynaecological oncology: a systematic review. BJOG : an international journal of obstetrics and gynaecology.

[8] Luckett T, King M, Butow P, Friedlander M, Paris T (2010). Assessing health-related quality of life in gynecologic oncology: a systematic review of questionnaires and their ability to detect clinically important differences and change. International journal of gynecological cancer : official journal of the International Gynecological Cancer Society.

[9] Boling W, Fouladi RT, Basen-Engquist K (2003). Health-related quality of life in gynecological oncology: instruments and psychometric properties. International journal of gynecological cancer : official journal of the International Gynecological Cancer Society.

[10] Vistad I, Fossa SD, Dahl AA (2006). A critical review of patient-rated quality of life studies of long-term survivors of cervical cancer. Gynecologic oncology.

[11] Pecorelli S (2009). Revised FIGO staging for carcinoma of the vulva, cervix, and endometrium. International journal of gynaecology and obstetrics: the official organ of the International Federation of Gynaecology and Obstetrics.

[12] Terwee CB, Bot SD, de Boer MR, van der Windt DA, Knol DL, Dekker J, Bouter LM, de Vet HC (2007). Quality criteria were proposed for measurement properties of health status questionnaires. Journal of clinical epidemiology.

[13] Hamoen EH, De Rooij M, Witjes JA, Barentsz JO, Rovers MM (2015). Measuring health-related quality of life in men with prostate cancer: A systematic review of the most used questionnaires and their validity. Urologic oncology.

[14] Greimel ER, Kuljanic Vlasic K, Waldenstrom AC, Duric VM, Jensen PT, Singer S, Chie W, Nordin A, Bjelic Radisic V, Wydra D (2006). The European Organization for Research and Treatment of Cancer (EORTC) Quality-of-Life questionnaire cervical cancer module: EORTC QLQ-CX24. Cancer.

[15] du Toit GC, Kidd M (2016). An analysis of the psychometric properties of the translated versions of the European Organisation for the Research and Treatment of Cancer QLQ CX24 questionnaire in the two South African indigenous languages of Xhosa and Afrikaans. European journal of cancer care.

[16] Hua CH, Guo HM, Guan XL, Kong FJ, Hou RJ, Zhang XY, Li SR (2013). Validation of the European Organization for Research and Treatment of Cancer cervical cancer module for Chinese patients with cervical cancer. Patient preference and adherence.

[17] Jayasekara H, Rajapaksa LC, Greimel ER (2008). The EORTC QLQ-CX24 cervical cancer-specific quality of life questionnaire: psychometric properties in a South Asian sample of cervical cancer patients. Psycho oncology.

[18] Shin DW, Ahn E, Kim YM, Kang S, Kim BG, Seong SJ, Cha SD, Park CY, Yun YH (2009). Cross-cultural application of the Korean version of the European Organization for Research and Treatment of Cancer quality of life questionnaire cervical cancer module. Oncology.

[19] Singer S, Kuhnt S, Momenghalibaf A, Stuhr C, Dimmel-Hennersdorf U, Kohler U, Einenkel J (2010). Patients' acceptance and psychometric properties of the EORTC QLQ-CX24 after surgery. Gynecologic oncology.

[20] Paradowska D, Tomaszewski KA, Balajewicz-Nowak M, Bereza K, Tomaszewska IM, Paradowski J, Pitynski K, Skotnicki P, Greimel ER, Bottomley A (2014). Validation of the Polish version of the EORTC QLQ-CX24 module for the assessment of health-related quality of life in women with cervical cancer. European journal of cancer care.

[21] Ding Y, Hu Y, Hallberg IR (2012). Psychometric properties of the Chinese version of the Functional Assessment of Cancer Therapy-Cervix (FACT-Cx) measuring health-related quality of life. Health and quality of life outcomes.

[22] Fernandes WC, Kimura M (2010). Health related quality of life of women with cervical cancer. Revista latino-americana de enfermagem.

[23] Fregnani CM, Fregnani JH, Dias de Oliveira Latorre Mdo R, de Almeida AM (2013). Evaluation of the psychometric properties of the Functional Assessment of Cancer Therapy-Cervix questionnaire in Brazil. PloS One.

