# Supplementary material for: Measuring health-related quality of life in cervical cancer patients: a systematic review of the most used questionnaires and their validity
Source: BMC Med Res Methodol. 2017 Jan 26;17:15. doi: 10.1186/s12874-016-0289-x (PMC5270308; doi:10.1186/s12874-016-0289-x)
Supplement: Additional file 1: — Search strategy systematic review represents the search strategy with combined synonyms for cervical cancer, questionnaires and quality of life in Pubmed, EMBASE and PsycINFO from inception to October 18th 2016. (DOCX 16 kb) [file 12874_2016_289_MOESM1_ESM.docx]

Appendix 1 Search strategy systematic review

Terms used for search strategy in: Pubmed, EMBASE and PsycINFO from inception to October 18^th^ 2016

| Domain: Cervical cancer | Determinant: Questionnaires | Outcome: Quality of life |
| --- | --- | --- |
| "Uterine Cervical Neoplasms"[Mesh] | "Surveys and Questionnaires"[Mesh] | “Quality of Life”[Mesh] |
| Synonyms or related terms in Title/Abstract | **Synonyms or related terms in Title/Abstract** | **Synonyms or related terms in Title/Abstract** |
| Cervical cancer | Questionnaire | Quality of Life |
| Cervical carcinoma | Self report | QOL |
| Cervical tumo(u)r | Outcome assessment | HRQOL |
| Cervical malignancy | Data collection | HR-QOL |
| Cervical neoplasm | Interview | QALY |
|  | Instrument | Patient-reported outcome |
|  | Survey |  |
|  | Tool |  |
|  | Evaluation |  |
|  | Measurement |  |

Mesh terms, synonyms and related terms were connected with OR. Domain, Determinant and Outcome were connected with AND.

**Pubmed:**
((((((((((("Uterine Cervical Neoplasms"[Mesh]) OR (“Carcinoma"[Mesh]) AND ("Cervix Uteri"[Mesh] OR cervi*[Tiab]))) OR ("Neoplasms"[Mesh] AND ("Cervix Uteri"[Mesh] OR cervi*[Tiab]))) OR (("Cervix Uteri"[Mesh] OR cervi*[Tiab]) AND cancer*[Tiab])) OR (("Cervix Uteri"[Mesh] OR cervi*[Tiab]) AND carcinoma*[Tiab])) OR (("Cervix Uteri"[Mesh] OR cervi*[Tiab]) AND tumour*[Tiab])) OR (("Cervix Uteri"[Mesh] OR cervi*[Tiab]) AND tumor*[Tiab])) OR (("Cervix Uteri"[Mesh] OR cervi*[Tiab]) AND malignan*[Tiab])) OR (("Cervix Uteri"[Mesh] OR cervi*[Tiab]) AND neoplas*[Tiab]))) AND (((((((((((((("Surveys and Questionnaires"[Mesh])) OR "Self Report"[Mesh]) OR "Outcome Assessment (Health Care)"[Mesh) OR "Patient Outcome Assessment"[Mesh]) OR "Data Collection"[Mesh]) OR "Interview, Psychological"[Mesh]) OR (Questionnair*[Tiab])) OR ((self[Tiab]) AND report*[Tiab]))) OR measur*[Tiab]) OR Tool* [Tiab]) OR Survey* [Tiab]) OR Interview*[Tiab]))) AND (((((((((“Quality of Life”[Mesh])) OR ((quality[Tiab] AND life[Tiab]))) OR (QOL[Tiab] OR HRQOL [Tiab] OR HR-QOL [Tiab] OR QALY [Tiab])) OR ((patient[Tiab] AND report*[Tiab] AND outcome*[Tiab]))) OR PRO[Tiab]) OR (((quality[Tiab] AND life[Tiab]) OR (QOL[Tiab] OR HRQOL[Tiab] OR HR-QOL[Tiab]) AND assess*[Tiab]))) OR (((quality[Tiab] AND life[Tiab]) OR (QOL[Tiab] OR HRQOL[Tiab] OR HR-QOL[Tiab]) AND evaluat*[Tiab])))))
