# Supplementary material for: Measuring health-related quality of life in cervical cancer patients: a systematic review of the most used questionnaires and their validity
Source: BMC Med Res Methodol. 2017 Jan 26;17:15. doi: 10.1186/s12874-016-0289-x (PMC5270308; doi:10.1186/s12874-016-0289-x)
Supplement: Additional file 2: — Data extraction form shows the format of the data extraction form used to systematically collect data per study on HRQoL tool, number of cervical cancer patients, and their respective FIGO stage. (DOCX 12 kb) [file 12874_2016_289_MOESM2_ESM.docx]

Appendix 2 Data extraction form

| **General data** | |
| --- | --- |
| Author |  |
| Title |  |
| Year of publication |  |
| Country |  |

| **HRQoL tools and patients** | | | |
| --- | --- | --- | --- |
| Tool(s) & n patients |  | | N= |
| Early stage |  | N= | |
| Advanced stage |  | N= | |

| Multiple HRQoL tools used |  |
| --- | --- |

| **Setting** |  |
| --- | --- |

| **Comments** |  |
| --- | --- |
