# Supplementary material for: Measuring health-related quality of life in cervical cancer patients: a systematic review of the most used questionnaires and their validity
Source: BMC Med Res Methodol. 2017 Jan 26;17:15. doi: 10.1186/s12874-016-0289-x (PMC5270308; doi:10.1186/s12874-016-0289-x)
Supplement: Additional file 3: — Search strategy validation studies represents the search strategy for validation studies with combined synonyms and terms for cervical cancer, validation studies/psychometrics and quality of life in Pubmed, EMBASE and PsycINFO from inception to October 18th 2016. (DOCX 12 kb) [file 12874_2016_289_MOESM3_ESM.docx]

Appendix 3 Search strategy validation studies

Terms used for search strategy in: Pubmed from inception to October 18^th^ 2016

| Domain: Cervical cancer | Determinant: Psychometrics | Outcome: Quality of life |
| --- | --- | --- |
| “Uterine Cervical Neoplasms”[Mesh] | “Psychometrics”[Mesh] | “Quality of Life”[Mesh] |
|  | “Validation Studies”[Mesh] |  |
|  | “Reproducibility of Results”[Mesh] |  |
| Synonyms or related terms in Title/Abstract | **Synonyms or related terms in Title/Abstract** | **Synonyms or related terms in Title/Abstract** |
| Cervi* | Psychometric* | Quality of Life |
|  | Validat* | QOL |
|  | Reliab* |  |

Mesh terms, synonyms and related terms were connected with OR. Domain, Determinant and Outcome were connected with AND.

(((("Uterine Cervical Neoplasms"[Mesh]) OR cervi*[Title/Abstract])) AND ((("Quality of Life"[Mesh]) OR quality of life[Title/Abstract]) OR qol[Title/Abstract])) AND (((((("Psychometrics"[Mesh]) OR "Validation Studies" [Publication Type]) OR "Reproducibility of Results"[Mesh]) OR validat*[Title/Abstract]) OR reliab*[Title/Abstract]) OR psychometric*[Title/Abstract])
