# Supplementary material for: Measuring health-related quality of life in cervical cancer patients: a systematic review of the most used questionnaires and their validity
Source: BMC Med Res Methodol. 2017 Jan 26;17:15. doi: 10.1186/s12874-016-0289-x (PMC5270308; doi:10.1186/s12874-016-0289-x)
Supplement: Additional file 4: — Adapted scoring model and scoring criteria shows the definition of the psychometric properties and the adapted scoring criteria from the COSMIN consortium which we applied to rate each cervical cancer specific HRQoL tool. (DOCX 16 kb) [file 12874_2016_289_MOESM4_ESM.docx]

Appendix 4 Adapted scoring model and scoring criteria.

| Property | Definition | Quality criteria^a,b^ |
| --- | --- | --- |
| 1. Content validity | The extent to which the domain of interest is comprehensively sampled by the items in the questionnaire | + A clear description is provided of the measurement aim, the target population, the concepts that are being measured, and the item selection AND target population and (investigators OR experts) were involved in item selection;  - No target population involvement;  ? A clear description of above-mentioned aspects is lacking OR only target population involved OR doubtful design or method;  X No information found on target population involvement. |
| 2. Internal  consistency | The extent to which items in a (sub)scale are intercorrelated, thus measuring the same construct | + Factor analyses performed on adequate sample size (7 * # items and >100) AND Cronbach’s alpha(s) calculated per dimension AND Cronbach’s alpha(s) between 0.70 and 0.95;  - Cronbach’s alpha(s) <0.70 or O0.95, despite adequate design and method;  ? No factor analysis OR doubtful design or method;  X No information found on internal consistency. |
| 3. Criterion validity | The extent to which scores on a particular questionnaire relate to a reference standard | + Convincing arguments that reference standard is ‘suitable’ AND correlation with reference standard ≥0.70;  - Correlation with reference standard <0.70, despite adequate design and method;  ? No convincing arguments that reference standard is ‘suitable’ OR doubtful design or method. Examples of not suitable reference standard: the prerequisite core questionnaire, non-HRQoL tools;  X No information found on criterion validity. |
| 4. Construct validity | The extent to which scores on a particular questionnaire relate to other measures (a reference measurement/result) in a manner that is consistent with theoretically derived hypotheses concerning the concepts that are being measured | + Specific hypotheses were formulated AND related to a ‘suitable’ reference standard AND at least 75% of the results are in accordance with these hypotheses. Depending on the hypothesis, the scores can be related to the prerequisite core questionnaire;  - Less than 75% of hypotheses were confirmed, despite adequate design and methods;  ? Doubtful design or method (e.g., no hypotheses, no suitable reference standard). Depending on the hypothesis, the prerequisite core questionnaire can be seen as not ‘suitable’;  X No information found on construct validity. |
| 5. Reproducibility |  |  |
| 5.1. Agreement | The extent to which the scores on repeated measures are close to each other (absolute measurement error) | + MIC<SDC OR MIC outside the LOA OR convincing arguments that agreement is acceptable;  - MIC≥SDC OR MIC equals or inside LOA, despite adequate design and method;  ? Doubtful design or method OR (MIC not defined AND no convincing arguments that agreement is acceptable);  X No information found on agreement. |
| 5.2. Reproducibility | The extent to which patients can be distinguished from each other, despite measurement errors (relative measurement error) | + ICC or weighted Kappa≥0.70;  - ICC or weighted Kappa<0.70, despite adequate design and method;  ? Doubtful design or method (e.g., time interval not mentioned, Kruskall Wallis, Wilcoxon, ANOVA, Mann Whitney, Cohen D, student T or other not suitable statistical tests,);  X No information found on reliability. |
| 6. Responsiveness | The ability of a questionnaire to detect clinically important changes over time | + SDC or SDC<MIC OR MIC outside the LOA OR RR > 1.96 OR AUC≥0.70;  - SDC or SDC≥MIC OR MIC equals or inside LOA OR RR≤1.96 OR AUC<0.70, despite? Doubtful design or method;  adequate design and methods;  X No information found on responsiveness. |
| 7. Floor- and ceiling  effects | The number of respondents who  achieved the lowest or highest possible  score | + ≤15% of the respondents achieved the highest or lowest possible scores;  - >15% of the respondents achieved the highest or lowest possible scores, despite adequate design and methods;  ? Doubtful design or method;  X No information found on floor and ceiling effects. |
| 8. Interpretability | The degree to which one can assign  qualitative meaning to quantitative  scores | + Mean and SD scores presented of at least four relevant subgroups of patients  and MIC defined;  ? Doubtful design or method OR less than four subgroups OR no MIC defined;  X No information found on interpretation and/or no subgroups. |
| MIC = minimal important change; SDC = smallest detectable change; LOA = limits of agreement; ICC = Intraclass correlation; SD = standard deviation.  a scores and ranking: + = positive rating (+1); - = negative rating (-1); ? = indeterminate rating (0); X = no information available (0).  b Doubtful design or method = lacking of a clear description of the design or methods of the study, sample size smaller than 50 subjects (should be at least 50 in every (subgroup) analysis), or any important methodological and/or statistical weakness in the design or execution of the study. | | |
