# Supplementary material for: Measuring health-related quality of life in cervical cancer patients: a systematic review of the most used questionnaires and their validity
Source: BMC Med Res Methodol. 2017 Jan 26;17:15. doi: 10.1186/s12874-016-0289-x (PMC5270308; doi:10.1186/s12874-016-0289-x)
Supplement: Additional file 5: — Supplemental references s1–s146 contains references to all individual studies included in the systematic review. (DOCX 37 kb) [file 12874_2016_289_MOESM5_ESM.docx]

Appendix 5 Supplemental references s1­–s146

1. Greenwald HP, McCorkle R, Baumgartner K, Gotay C, Neale AV. Quality of life and disparities among long-term cervical cancer survivors. J Cancer Surviv 2014;8(3):419-26.
2. Gotay CC, Farley JH, Kawamoto CT, Mearig A. Adaptation and quality of life among long-term cervical cancer survivors in the military health care system. Mil Med 2008;173(10):1035-41.
3. Vistad I, Cvancarova M, Kristensen GB, Fossa SD. A study of chronic pelvic pain after radiotherapy in survivors of locally advanced cervical cancer. J Cancer Surviv 2011;5(2):208-16.
4. Sekse RJT, Hufthammer KO, Vika ME. Fatigue and quality of life in women treated for various types of gynaecological cancers: a cross-sectional study. J Clin Nurs 2015;24(3-4):546-55.
5. Hazewinkel MH, Sprangers MAG, van der Velden J, Burger MPM, Roovers JPWR. Severe pelvic floor symptoms after cervical cancer treatment are predominantly associated with mental and physical well-being and body image: A cross-sectional study. Int J Gynecol Cancer 2012;22(1):154-60.
6. Capelli G, De Vincenzo RI, Addamo A, Bartolozzi F, Braggio N, Scambia G. Which dimensions of health-related quality of life are altered in patients attending the different gynecologic oncology health care settings? Cancer 2002;95(12):2500-7.
7. Distefano M, Riccardi S, Capelli G, Costantini B, Petrillo M, Ricci C, et al. Quality of life and psychological distress in locally advanced cervical cancer patients administered pre-operative chemoradiotherapy. Gynecol Oncol 2008;111(1):144-50.
8. Seibaek L, Petersen LK. Self-valued health in Danish women after cervix cancer surgery--A retrospective questionnaire study. Eur J Oncol Nurs 2007;11(2):183-6.
9. Xie Y, Zhao FH, Lu SH, Huang H, Pan XF, Yang CX, et al. Assessment of quality of life for the patients with cervical cancer at different clinical stages. Chin J Cancer 2013;32(5):275-82.
10. Brotto LA, Heiman JR, Goff B, Greer B, Lentz GM, Swisher E, et al. A psychoeducational intervention for sexual dysfunction in women with gynecologic cancer. Arch Sex Behav 2008;37(2):317-29.
11. Crevenna R, Cenik F, Galle A, Komanadj TS, Keilani M. Feasibility, acceptance and long-term exercise behaviour in cancer patients: an exercise intervention by using a swinging-ring system. Wien Klin Wochenschr 2015;127(19-20):751-5.
12. Beatty LJ, Adams J, Sibbritt D, Wade TD. Evaluating the impact of cancer on complementary and alternative medicine use, distress and health related QoL among Australian women: A prospective longitudinal investigation. Complement Ther Med 2012;20(1-2):61-69.
13. Perwitasari DA, Atthobari J, Mustofa M, Dwiprahasto I, Hakimi M, Gelderblom H, et al. Impact of chemotherapy-induced nausea and vomiting on quality of life in Indonesian patients with gynecologic cancer. Int J Gynecol Cancer 2012;22(1):139-45.
14. Adamsen L, Quist M, Andersen C, Moller T, Herrstedt J, Kronborg D et al. Effect of a multimodal high intensity exercise intervention in cancer patients undergoing chemotherapy: randomised controlled trial. BMJ Online 2009;339**:**b3410.
15. Bradley S, Rose S, Lutgendorf S, Costanzo E, Anderson B. Quality of life and mental health in cervical and endometrial cancer survivors. Gynecol Oncol 2006;100(3):479-86.
16. Visser MRM, van Lanschot JJB, van der Velden J, Kloek JJ, Gouma DJ, Sprangers MAG. Quality of life in newly diagnosed cancer patients waiting for surgery is seriously impaired. J Surg Oncol 2006;93(7):571-7.
17. Wenzel L, Dogan-Ates A, Habbal R, Berkowitz R, Goldstein DP, Bernstein M, et al. Defining and measuring reproductive concerns of female cancer survivors. J Natl Cancer Inst Monogr 2005;34:94-8.
18. Le Borgne G, Mercier M, Woronoff AS, Guizard AV, Abeilard E, Caravati-Jouvenceaux A, et al. Quality of life in long-term cervical cancer survivors: A population-based study. Gynecol Oncol 2013;129:222-8.
19. Caixeta GA, Castro EEC, Silva-Filho L, Reis FM, Cunha-Melo JR, Triginelli SA. Quality of life and mental health in Brazilian women treated for invasive carcinoma of the cervix. Int J Gynecol Cancer 2014;24(4):794-9.
20. Kent EE, Ambs A, Mitchell SA, Clauser SB, Smith AW, Hays RD. Health-related quality of life in older adult survivors of selected cancers: data from the SEER-MHOS linkage. Cancer 2015;121(5):758-65.
21. Korfage IJ, Essink-Bot ML, Mols F, van de Poll-Franse L, Kruitwagen R, van Ballegooijen M. Health-related quality of life in cervical cancer survivors: a population-based survey. Int J Radiat Oncol Biol Phys 2009;73(5):1501-9.
22. Hung MC, Wu CL, Hsu YY, Hwang JS, Cheng YM, Wang JD. Estimation of potential gain in quality of life from early detection of cervical cancer. Value Health 2014;17(4):482-6.
23. Pasek M, Urbanksi K, Suchocka L. Quality of life in advanced cervical cancer patients subjected to radiotherapy-A WHOQOL BREF questionnaire study. Psycho-Oncol 2013;7(2):107-12.
24. Nuhu FT, Adebayo KO, Adejumo O. Quality of life of people with cancers in Ibadan, Nigeria. J Ment Health 2013;22(4):325-33.
25. Awadalla AW, Ohaeri JU, Gholoum A, Khalid AOA, Hamad HMA, Jacob A. Factors associated with quality of life of outpatients with breast cancer and gynecologic cancers and their family caregivers: a controlled study. BMC Cancer 2007;7(102).
26. Ceccaroni M, Roviglione G, Spagnolo E, Casadio P, Clarizia R, Peiretti M, et al. Pelvic dysfunctions and quality of life after nerve-sparing radical hysterectomy: a multicenter comparative study. Anticancer Res 2012;32(2):581-8.
27. Jorge LLR, da Silva SR. Evaluation of the quality of life of gynecological cancer patients submitted to antineoplastic chemotherapy. Rev Lat Am Enfermagem 2010;18(5):849-55.
28. Vaz AF, Pinto-Neto AM, Conde DM, Coast-Paiva L, Morais SS, Pedro AO, et al. Quality of life and menopausal and sexual symptoms in gynecologic cancer survivors: a cohort study. Menopause 2011;18(6):662-9.
29. Grion RC, Baccaro LF, Vaz AF, Costa-Paiva L, Conde DM, Pinto-Neto AM. Sexual function and quality of life in women with cervical cancer before radiotherapy: a pilot study. Arch Gynecol Obstet 2016;293(4):879-86.
30. Lai BPY, Tang CS, Chung TKH. Age-specific correlates of quality of life in Chinese women with cervical cancer. Support Care Cancer 2009;17(3):271-8.
31. Bouhnik AD, Bendiane MK, Cortaredona S, Teyssier LS, Rey D, Berenger C, et al. The labour market, psychosocial outcomes and health conditions in cancer survivors: Protocol for a nationwide longitudinal survey 2 and 5 years after cancer diagnosis (the VICAN survey). BMJ Open 2015;5(3):e005971.
32. Ashing-Giwa KT, Lim J, Tang G. Surviving cervical cancer: Does health-related quality of life influence survival? Gynecol Oncol 2010;118(1):35-42.
33. Ashing-Giwa KT, Tejero JS, Kim J, Padilla GV, Kagawa-Singer M, Tucker MB, et al. Cervical cancer survivorship in a population based sample. Gynecol Oncol 2009;112(2):358-64.
34. Pilger A, Richter R, Fotopoulou C, Beteta C, Klapp C, Sehouli, A. Quality of life and sexuality of patients after treatment for gynaecological malignancies: results of a prospective study in 55 patients. Anticancer Res 2012;32(11):5045-9.
35. Frumovitz M, Sun CC, Schover LR, Munsell MF, Jhingran A, Wharton JT, et al. Quality of life and sexual functioning in cervical cancer survivors. J Clin Oncol 2005;23(30):7428-36.
36. Canada AL, Schover LR. The psychosocial impact of interrupted childbearing in long-term female cancer survivors. Psychooncology 2012;21(2):134-43.
37. Levin AO, Carpenter KM, Fowler JM, Brothers BM, Andersen BL, Maxwell GL. Sexual morbidity associated with poorer psychological adjustment among gynecological cancer survivors. Int J Gynecol Cancer 2010;20(3):461-470.
38. Fleming ND, Ramirez PT, Soliman PT, Schmeler KM, Chisholm GB, Nick AM, et al. Quality of life after radical trachelectomy for early-stage cervical cancer: A 5-year prospective evaluation. Gynecol Oncol 2016;S0090-8258(16)31484-6.
39. Lee JA, Kim SY, Kim Y, Oh J, Kim HJ, Jo DY, et al. Comparison of health-related quality of life between cancer survivors treated in designated cancer centers and the general public in Korea. Jpn J Clin Oncol 2014;44(2):141-52.
40. Lee SY, Kim SJ, Shin J, Han KT, Park EC. The impact of job status on quality of life: General population versus long-term cancer survivors. Psychooncology 2015;24(11):1552-9.
41. Endarti D, Riewpaiboon A, Thavorncharoensap M, Praditsitthikorn N, Hutubessy R, Kristina SA. Evaluation of Health-Related Quality of Life among Patients with Cervical Cancer in Indonesia. Asian Pac J Cancer Prev 2015;16(8):3345-50.
42. Zhao ZM, Pan XF, Lv SH, Xie Y, Zhang SK, Qiao YL. Quality of life in women with cervical precursor lesions and cancer: a prospective, 6-month, hospital-based study in China. Chin J Cancer 2014;33(7):339-45.
43. Lang HC, Chang K, Ying YH. Quality of life, treatments, and patients' willingness to pay for a complete remission of cervical cancer in Taiwan. Health Econ 2012;21(10):1217-33.
44. Kimman M, Jan S, Monaghan H, Woodward M. The relationship between economic characteristics and health-related quality of life in newly diagnosed cancer patients in Southeast Asia: results from an observational study. Qual Life Res 2015;24(4):937-49.
45. Weaver KE, Forsythe LP, Reeve BB, Alfano CM, Rodriguez JL, Sabatino SA, et al. Mental and physical health-related quality of life among U.S. cancer survivors: population estimates from the 2010 National Health Interview Survey. Cancer Epidemiol Biomarkers Prev 2012;21(11):2108-17.
46. Osann K, Hsieh S, Nelson EL, Monk BJ, Chase D, Cella D, et al. Factors associated with poor quality of life among cervical cancer survivors: implications for clinical care and clinical trials. Gynecol Oncol 2014;135(2):266-72.
47. Doll KM, Kalinowski AK, Snavely AC, Irwin DE, Bensen JT, Bae-Jump VL, et al. Obesity is associated with worse quality of life in women with gynecologic malignancies: an opportunity to improve patient-centered outcomes. Cancer 2015;121(3):395-402.
48. Wenzel L, Osann K, Hsieh S, Tucker JA, Monk BJ, Nelson EL. Psychosocial telephone counseling for survivors of cervical cancer: results of a randomized biobehavioral trial. J Clin Oncol 2015;33(10):1171-9.
49. Greimel E, Thiel I, Peintinger F, Cegnar I, Pongratz E. Prospective assessment of quality of life of female cancer patients. Gynecol Oncol 2002;85(1):140-7.
50. Greimel ER, Freidl W. Functioning in daily living and psychological well-being of female cancer patients. J Psychosom Obstet Gynaecol 2000;21(1):25-30.
51. Frazier LM, Miller VA, Horbelt V, Delmore JE, Miller BE, Averett EP. Employment and quality of survivorship among women with cancer: Domains not captured by quality of life instruments. Cancer Control 2009;16(1):57-65.
52. Tornatta JM, Carpenter JS, Schilder J, Cardenes HR. Representations of vaginal symptoms in cervical cancer survivors. Cancer Nurs 2009;32(5):378-84.
53. Carter JR, Dwight Chen M, Fowler JM, Carson LF, Twiggs LB. The effect of prolonged cycles of chemotherapy on quality of life in gynaecologic cancer patients. J Obstet Gynaecol Res 1997;23(2):197-203.
54. Von Gruenigen VE, Coleman RL, Li AJ, Heard MC, Miller DS, Hemsell DL. Bacteriology and treatment of malodorous lower reproductive tract in gynecologic cancer patients. Obstet Gynecol 2000;96(1):23-7.
55. Sood AK, Nygard I, Shahin MS, Sorosky JI, Lutgendorf SK, Rao SSC. Anorectal dysfunction after surgical treatment for cervical cancer. J Am Coll Surg 2002;195(4):513-9.
56. Miller BE, Pittman RN, Case D, McQuellon RP. Quality of life after treatment for gynecologic malignancies: a pilot study in an outpatient clinic. Gynecol Oncol 2002;87(2):178-84.
57. Taechaboonsermsak P, Kaewkungwal J, Singhasivanon P, Fungladda W, Wilailak S. Causal relationship between health promoting behavior and quality of life in cervical cancer patients undergoing radiotherapy. Southeast Asian J Trop Med Public Health 2005;36(6):1568-75.
58. Taneepanichskul S, Lertmaharit S, Pongpanich S, Termrungruanglert W, Havanond P, Khemapech N, et al. Quality of life among thai women diagnosed with cervical cancer and cervical intraepithelial neoplasia at King Chulalongkorn Memorial Hospital. J Med Assoc Thai 2011;94(8):902-7.
59. Wilailak S, Lertkhachonsuk A, Lohacharoenvanich N, Luengsukcharoen SC, Jirajaras M, Likitanasombat P, et al. Quality of life in gynecologic cancer survivors compared to healthy check-up women. J Gynecol Oncol 2011;22(2):103-9.
60. Rithirangsriroj K, Manchana T, Akkayagorn L. Efficacy of acupuncture in prevention of delayed chemotherapy induced nausea and vomiting in gynecologic cancer patients. Gynecol Oncol 2015;136(1): 82-6.
61. Asuzu CC, Elumelu TN. Assessing cancer patients' quality of life and coping mechanisms in Radiotherapy Department of the University College Hospital, Ibadan. Psychooncology 2013;22(10):2306-12.
62. Bae H, Park H. Sexual function, depression, and quality of life in patients with cervical cancer. Support Care Cancer 2016;24(3):1277-83.
63. Kobayashi M, Ohno T, Noguchi W, Matsuda A, Matsushima E, Kato S, et al. Psychological distress and quality of life in cervical cancer survivors after radiotherapy: do treatment modalities, disease stage, and self-esteem influence outcomes? Int J Gynecol Cancer 2009;19(7):1264-8.
64. Hockel M, Schlenger K, Hamm H, Knapstein PG, Hohenfeilner R, Rosler HP. Five-year experience with combined operative and radiotherapeutic treatment of recurrent gynecologic tumors infiltrating the pelvic wall. Cancer 1996;77(9):1918-33.
65. Beesley VL, Eakin EG, Janda M, Battistutta D. Gynecological cancer survivors' health behaviors and their associations with quality of life. Cancer Causes Control 2008;19(7):775-82.
66. Laky B, Jnda M, Kondalsamy-Chennakesavan S, Cleghorn G, Obermair A. Pretreatment malnutrition and quality of life - association with prolonged length of hospital stay among patients with gynecological cancer: A cohort study. BMC Cancer 2010;10(232).
67. Juraskova I, Bonner C, Bell ML, Sharpe L, Robertson R, Butow P. Quantity vs. quality: an exploration of the predictors of posttreatment sexual adjustment for women affected by early stage cervical and endometrial cancer. J Sex Med 2012;9(11):2952-60.
68. Stafford L, Foley E, Judd F, Gibson P, Kiropoulos L, Couper J. Mindfulness-based cognitive group therapy for women with breast and gynecologic cancer: A pilot study to determine effectiveness and feasibility. Support Care Cancer 2013;21(11):3009-19.
69. Penson RT, Huang HQ, Wenzel LB, Monk BJ, Stockman S, Long HJ 3rd, et al. Bevacizumab for advanced cervical cancer: patient-reported outcomes of a randomised, phase 3 trial (NRG Oncology-Gynecologic Oncology Group protocol 240). Lancet Oncol 2015;16(3):301-11.
70. Monk BJ, Sill MW, McMeekin S, Cohn DE, Ramondetta LM, Boardman CH, et al. Phase III trial of four cisplatin-containing doublet combinations in stage IVB, recurrent, or persistent cervical carcinoma: a Gynecologic Oncology Group study. J Clin Oncol 2009;27(28):4649-55.
71. Carter J, Sonoda Y, Baser RE, Raviv L, Chi DS, Barakat RR, et al. A 2-year prospective study assessing the emotional, sexual, and quality of life concerns of women undergoing radical trachelectomy versus radical hysterectomy for treatment of early-stage cervical cancer. Gynecol Oncol 2010;119(2):358-65.
72. Monk BJ, Huang HQ, Cella D, Long HJ 3rd. Quality of life outcomes from a randomized phase III trial of cisplatin with or without topotecan in advanced carcinoma of the cervix: a Gynecologic Oncology Group Study. J Clin Oncol 2005;23(21):4617-25.
73. Gupta S, Singh PK, Bisth SS, Bhatt ML, Pant MC, Gupta R, et al. Role of recombinant human erythropoietin in patients of advanced cervical cancer treated by chemoradiotherapy. Cancer Biol Ther 2009;8(1):13-7.
74. Lutgendorf SK, Mullen-Houser E, Russell D, DeGeest K, Jacobson G, Hart L, et al. Preservation of immune function in cervical cancer patients during chemoradiation using a novel integrative approach. Brain Behav Immun 2010;24(8):1231-40.
75. Adams E, Boulton MG, Horne A, Rose PW, Durrant L, et al. The Effects of Pelvic Radiotherapy on Cancer Survivors: Symptom Profile, Psychological Morbidity and Quality of Life. Clin Oncol 2014;26(1):10-7.
76. Goker A, Guvenal T, Yanikkerem E, Turhan A, Koyuncu FM. Quality of life in women with gynecologic cancer in Turkey. Asian Pac J Cancer Prev 2011;12(11):3121-8.
77. Hsu LN, Lin SE, Luo HL, Chang JC, Chiang PH. Double-barreled colon conduit and colostomy for simultaneous urinary and fecal diversions: long-term follow-up. Ann Surg Oncol 2014;21**:**S522-7.
78. Li CC, Chen ML, Chang TC, Chou HH, Chen MY. Social support buffers the effect of self-esteem on quality of life of early-stage cervical cancer survivors in Taiwan. Eur J Oncol Nurs 2015;19(5):486-94.
79. Ahn WS, Kim DJ, Chae GT, Lee JM, Bae SM, Sin JI et al. Natural killer cell activity and quality of life were improved by consumption of a mushroom extract, Agaricus blazei Murill Kyowa, in gynecological cancer patients undergoing chemotherapy. Int J Gynecol Cancer 2004;14(4):589-94.
80. Masika GM, Wettergren L, Kohi TW, von Essen L. Health-related quality of life and needs of care and support of adult Tanzanians with cancer: A mixed-methods study. Health Qual Life Outcomes 2012;10(133).
81. Berveling MJ, Langendijk JA, Beukema JC, Mourits MJE, Reyners AKL, Pras E. Health-related quality of life and late morbidity in concurrent chemoradiation and radiotherapy alone in patients with locally advanced cervical carcinoma. J Gynecol Oncol 2011;22(3):152-60.
82. Hinnen C, Pool G, Holwerda N, Sprangers M, Sanderman R, Hagedoorn M. Lower levels of trust in one's physician is associated with more distress over time in more anxiously attached individuals with cancer. Gen Hosp Psychiatry 2014;36(4):382-7.
83. Bye A, Trope C, Loge JH, Hjermstad M, Kaasa A. Health-related quality of life and occurrence of intestinal side effects after pelvic radiotherapy: Evaluation of long-term effects of diagnosis and treatment. Acta Oncol 2000;39(2):173-80.
84. Rustoen T, Fossa SD, Skarstein J, Moum T. The impact of demographic and disease-specific variables on pain in cancer patients. J Pain Symptom Manage 2003;26(2):696-704.
85. Pasek M, Suchocka L, Urbanski K. Quality of life in cervical cancer patients treated with radiation therapy. J Clin Nurs 2013;22(5-6):690-7.
86. Kim SI, Lim MC, Lee JS, Lee Y, Park KB, Joo J, et al. Impact of lower limb lymphedema on quality of life in gynecologic cancer survivors after pelvic lymph node dissection. Eur J Obstet Gynecol Reprod Biol 2015;192:31-6.
87. Kamau RK, Osotu AO, Njuguna EM. Effect of diagnosis and treatment of inoperable cervical cancer on quality of life among women receiving radiotherapy at Kenyatta National Hospital. East Afr Med J 2007;84(1):24-30.
88. Scheele J, Niazi F, Drevs J, Diergarten K, Toure P, J. A pilot study of Auron Misheil Therapy in patients with advanced cervical cancer: tumor response and its correlation with clinical benefit response, and preliminary quality of life data. Oncol Rep 2009;22(4):877-83.
89. Grimm D, Hasenburg A, Eulenburg C, Steinsiek L, Mayer S, Eltrop S, et al. Sexual Activity and Function in Patients With Gynecological Malignancies After Completed Treatment. Int J Gynecol Cancer 2015;25(6):1134-41.
90. Tadele N. Evaluation of quality of life of adult cancer patients attending Tikur Anbessa specialized referal hospital, Addis Ababa Ethiopia. Ethiop J Health Sci 2015;25(1):53-62.
91. Xie BG, Lu WY, Huang YH, Zhu WJ. Quality of life in cervical cancer treated with systematic nerve-sparing and modified radical hysterectomies. J Obstet Gynaecol 2015;35(8):839-43.
92. Gamper EM, Giesinger JM, Oberguggenberger A, Kemmler G, Wintner LM, Gattringer K, et al. Taste alterations in breast and gynaecological cancer patients receiving chemotherapy: Prevalence, course of severity, and quality of life correlates. Acta Oncol 2012;51(4):490-6.
93. Hawighorst-Knapstein S, Fusshoeller C, Franz C, Trautmann K, Schmidt M, Pilch H, et al. The impact of treatment for genital cancer on quality of life and body image--results of a prospective longitudinal 10-year study. Gynecol Oncol 2004;94(2):398-403.
94. Hsu WC, Chung NN, Chen YC, Tin LL, Wang PM, Hsieh PC, et al. Comparison of surgery or radiotherapy on complications and quality of life in patients with the stage IB and IIA uterine cervical cancer. Gynecol Oncol 2009;115(1):41-5.
95. Klee M, Thranov I, Machin D. Life after radiotherapy: the psychological and social effects experienced by women treated for advanced stages of cervical cancer. Gynecol Oncol 2000;76(1):5-13.
96. Minig L, Ivan Velez J, Trimble EL, Biffi R, Maggioni A, Jeffery DD. Changes in short-term health-related quality of life in women undergoing gynecologic oncologic laparotomy: an associated factor analysis. Support Care Cancer 2013;21(3):715-26.
97. De Paula LCL, Fonseca F, Perazzo F, Cruz FM, Cubero D, Trufelli DC, et al. Uncaria tomentosa (Cat's Claw) improves quality of life in patients with advanced solid tumors. J Altern Complement Med 2015;21(1):22-30.
98. Li J, Humphreys K, Eriksson M, Dar H, Brandberg Y, Hall P, et al. Worse quality of life in young and recently diagnosed breast cancer survivors compared with female survivors of other cancers: A cross-sectional study. Int J Cancer 2016;139(11):2415-25.
99. Song EK, Shim H, Han HS, Sun D, Lee SI, Kang MH, et al. A prospective multicentre study to evaluate the efficacy and tolerability of osmotic release oral system (oros®) hydromorphone in opioid-naive cancer patients: results of the Korean south West oncology group study. Pain Res Manag 2015;20(6):293-299.
100. Nazik E, Nazik H, Ozdemir F, Soydan S. Social support and quality of life in Turkish patients with gynecologic cancer. Asian Pac J Cancer Prev 2014;15(7):3081-6.
101. Nuhu F, Odejide OA, Adebayo KO, Yusuf AJ. Psychological and physical effects of pain on cancer patients in Ibadan, Nigeria. Afr J Psychiatry 2009;12(1):64-70.
102. Padilla GV, Mishel MH, Grant MM. Uncertainty, appraisal and quality of life. Qual Life Res 1992;1(3):155-65.
103. Damodar G, Gopinath S, Vijayakumar S, Rao AY. Reasons for low quality of life in South Indian cancer patient population: a prospective observational study. Indian J Pharm Sci 2014;76(1):2-9.
104. Yavas G, Dogan NU, Yavas C, Benzer N, Yuce D, Celik C. Prospective assessment of quality of life and psychological distress in patients with gynecologic malignancy: a 1-year prospective study. Int J Gynecol Cancer 2012;22:1096-1101.
105. Symonds RP, Davidson SE, Chan S, Reed NS, McMahon T, Rai D, et al. SCOTCERV: a phase II trial of docetaxel and gemcitabine as second line chemotherapy in cervical cancer. Gynecol Oncol 2011;123(1):105-9.
106. Barnas E, Skret-Magierlo J, Skret A, Bidzinski M. The quality of life of women treated for cervical cancer. Eur J Oncol Nurs 2012;16(1):59-63.
107. Barnas E, Borowiec-Domka E, Trawinska J, Ciepiela I, Ras R, Pasierb D, et al. Quality of life of women treated for cervical cancer in Rzeszow. Curr Gynecol Oncol 2013;11(3):183-90.
108. du Toit GC, Kidd M. Prospective Quality of Life Study of South African Women Undergoing Treatment for Advanced-stage Cervical Cancer. Clin Ther 2015;37(10):2324-31.
109. Yoo SH, Yun YH, Park S, Kim YA, Park SY, Bay DS, et al. The correlates of unemployment and its association with quality of life in cervical cancer survivors. J Gynecol Oncol 2013;24(4):367-75.
110. Azmawati MN, Najibah E, Hatta MDAZ, Norfazilah A. Quality of life by stage of cervical cancer among Malaysian patients. Asian Pac J Cancer Prev 2014;15(13):5283-6.
111. Fuchs-Tarlovsky V, Rivera MAC, Altamirano KA, Lopez-Alvarenga JC, Ceballos-Reyes GM. Antioxidant supplementation has a positive effect on oxidative stress and hematological toxicity during oncology treatment in cervical cancer patients. Support Care Cancer 2013;21(5):1359-63.
112. Bifulco G, De Rosa N, Piccoli R, Bertrando A, Lavitola G, Morra I, et al. Quality of life, lifestyle behavior and employment experience: A comparison between young and midlife survivors of gynecology early stage cancers. Gynecol Oncol 2012;124(3):444-51.
113. Ferrandina G, Mantegna G, Petrillo M, Fuoco G, Venditti L, Terzano S, et al. Quality of life and emotional distress in early stage and locally advanced cervical cancer patients: A prospective, longitudinal study. Gynecol Oncol 2012;124(3):389-94.
114. Pisani C, Deantonio L, Surico D, Brambilla M, Galla A, Ferrara E, et al. Quality of life in patients treated by adjuvant radiotherapy for endometrial and cervical cancers: correlation with dose-volume parameters. Clin Transl Oncol 2015 Nov 25. [Epub ahead of print]. DOI 10.1007/s12094-015-1458-9
115. Gargiulo P, Arenare L, Pisano C, Cecere SC, Falivene S, Greggi S, et al. Long-Term Toxicity and Quality of Life in Patients Treated for Locally Advanced Cervical Cancer. Oncology 2016;90(1):29-35.
116. Shin DW, Nam JH, Kwon YC, Park SY, Bae DS, Park CT, et al. Comorbidity in disease-free survivors of cervical cancer compared with the general female population. Oncology 2008;74(3-4):207-15.
117. Kumar S, Rana ML, Verma K, Singh N, Sharma AK, Maria AK, et al. PrediQt-Cx: Post treatment health related quality of life prediction model for cervical cancer patients. PLoS One 2014;9(2):e89851.
118. Torkzahrani S, Rastegari L, Khodakarami N, Akbarzadeh-Baghian A, Alizadeh K. Quality of life and its related factors among Iranian cervical cancer survivors. Iran Red Crescent Med J 2013;15(4):320-3.
119. Plotti F, Sansone M, Di Donato V, Antonelli E, Altavilla T, Angioli R, et al. Quality of life and sexual function after type C2/type III radical hysterectomy for locally advanced cervical cancer: a prospective study. J Sex Med 2011;8(3):894-904.
120. Sowa E, Kuhnt S, Hinz A, Schroder C, Deutsch T, Geue K. Postoperative health-related quality of life of cervical cancer patients - A comparison between the Wertheim-Meigs operation and Total Mesometrial Resection (TMMR). Geburtsh Frauenheilk 2014;74(7):670-6.
121. Krikeli M, Ekonomopoulou MT, Tzitzikas I, Goutzioulis A, Mystakidou K, Pistevou-Gombaki K. Comparison of the impact of radiotherapy and radiochemotherapy on the quality of life of 1-year survivors with cervical cancer. Cancer Manag Res 2011;3:247-51.
122. Holt KA, Mogensen O, Jensen PT, Hansen DG. Goal setting in cancer rehabilitation and relation to quality of life among women with gynaecological cancer. Acta Oncol 2015;54(10):1814-23.
123. Froding LP, Ottosen C, Mosgaard BJ, Jensen PT. Quality of life, urogynecological morbidity, and lymphedema after radical vaginal trachelectomy for early-stage cervical cancer. Int J Gynecol Cancer 2015;25(4):699-706.
124. Baffert S, Alran S, Fourchotte V, Traore MA, Simondi C, Mathevet P, et al. Laparoscopic hysterectomy after concurrent radiochemotherapy in locally advanced cervical cancer compared to laparotomy: A multi institutional prospective pilot study of cost, surgical outcome and quality of life. Eur J Surg Oncol 2016;42(3):391-9.
125. Li S, Hu T, Chen Y, Zhou H, Li X, Cheng X, et al. Adjuvant chemotherapy, a valuable alternative option in selected patients with cervical cancer. PLoS One 2013;8(9):e73837.
126. Ye S, Yang J, Cao D, Zhu L, Lang J, Chuang LT, et al. Quality of life and secual function of patients following radical hysterectomy and vaginal extension. J Sex Med 2014;11:1334–42.
127. Greimel ER, Winter R, Kapp KS, Haas J. Quality of life and sexual functioning after cervical cancer treatment: a long-term follow-up study. Psychooncology 2009;18(5):476-82.
128. Kirchheiner K, Nout R, Lindegaard J, Limbergen EV, Jurgenliemk-Schulz IM, Haie-Meder C, et al. Do clinicians and patients agree regarding symptoms? A comparison after definitive radiochemotherapy in 223 uterine cervical cancer patients. Strahlenther Onkol 2012;188(10):933-9.
129. Kirchheiner K, Czajka-Pepl A, Ponocny-Seliger E, Scharbert G, Wetzel L, Nour RA, et al. Posttraumatic stress disorder after high-dose-rate brachytherapy for cervical cancer with 2 fractions in 1 application under spinal/epidural anesthesia: incidence and risk factors. Int J Radiat Oncol Biol Phys 2014;89(2):260-7.
130. Bjelic-Radisic V, Jensen PT, Kuljanic Vlasic K, Waldenstrom A, Singer S, Chie W, et al. Quality of life characteristics inpatients with cervical cancer. Eur J Cancer 2012;48(16):3009-18.
131. Ljuca D, Marosevic G. Quality of life in patients with cervical cancer FIGO IIb stage after concomitant chemoradiotherapy. Radiol Oncol 2009;43(4):293-8.
132. Lee Y, Lim MC, Kim SI, Joo J, Lee DO, Park SY. Comparison of quality of life and sexuality between cervical cancer survivors and healthy women. Cancer Res Treat 2016;48(4):1321-1329.
133. Khalil J, Bellefqih S, Sahli N, Afif M, Elkacemi H, Elmajjaoui S, et al. Impact of cervical cancer on quality of life: beyond the short term (Results from a single institution): Quality of life in long-term cervical cancer survivors: results from a single institution. Gynecol Oncol Res Pract 2015;2:7.
134. Derks M, van der Velden J, Frijstein MM, Vermeer WM, Stiggelbout AM, Roovers JP, et al. Long-term pelvic floor function and quality of life after radical surgery for cervical cancer: a multicenter comparison between different techniques for radical hysterectomy with pelvic lymphadenectomy. Int J Gynecol Cancer 2016;26(8):1538-43.
135. Dahiya N, Acharya AS, Bachani D, Sharma D, Gupta S, Haresh K, et al. Quality of life of patients with advanced cervical cancer before and after chemoradiotherapy. Asian Pac J Cancer Prev 2016;17(7):3095-9.
136. Zaid T, Burzawa J, Basen-Enquist K, Bodurka DC, Ramondetta LM, Brwon J, et al. Use of social media to conduct a cross-sectional epidemiologic and quality of life survey of patients with neuroendocrine carcinoma of the cervix: A feasibility study. Gynecol Oncol 2014;132:149-53.
137. Chase DM, Kauderer J, Wenzel L, Ramondetta L, Cella D, Long HJ 3rd, et al. Factors associated with grade 3 or 4 treatment-related toxicity in women with advanced or recurrent cervical cancer: an exploratory analysis of NRG Oncology/Gynecologic Oncology Group trials 179 and 204. Int J Gynecol Cancer 2015;25(2):303-8.
138. Downs Jr LS, Chura JC, Argenta PA, Judson PL, Ghebre R, Geller MA, et al. Ifosfamide, paclitaxel, and carboplatin, a novel triplet regimen for advanced, recurrent, or persistent carcinoma of the cervix: A phase II trial. Gynecol Oncol 2011;120(2):265-9.
139. Nelson EL, Wenzel LB, Osann K, Dogan-Ates A, Chantana N, Reina-Patton A, et al. Stress, immunity, and cervical cancer: biobehavioral outcomes of a randomized clinical trial. Clin Cancer Res 2008;14(7):2111-8.
140. Moore DH, Blessing JA, McQuellon RP, Thaler HT, Cella D, Benda J, et al. Phase III study of cisplatin with or without paclitaxel in stage IVB, recurrent, or persistent squamous cell carcinoma of the cervix: a gynecologic oncology group study. J Clin Oncol 2004;22(15):3113-9.
141. Jenkins AD, Ramondetta LM, Sun C, Johnston T, Wolf JK, Bodurka DC, et al. Phase II trial of capecitabine in recurrent squamous cell carcinoma of the cervix. Gynecol Oncol 2005;97(3):840-4.
142. Ditto A, Martinelli F, Borreani C, Kusamura S, Hazonet F, Brunelli C, et al. Quality of life and sexual, bladder, and intestinal dysfunctions after class III nerve-sparing and class II radical hysterectomies: A questionnaire-based study. Int J Gynecol Cancer 2009;19(5):953-7.
143. Ding Y, Hu Y, Hallberg IR. Health-related quality of life and associated factors in Chinese women with cervical cancer: a 9-month follow-up. Cancer Nurs 2013;36(4):E18-26.
144. Nie SX, Gao CQ. Health behaviors and quality of life in Chinese survivors of cervical cancer: a retrospective study. Onco Targets Ther 2014;7:627-32.
145. Li J, Huang J, Zhang J, Li Y. A home-based, nurse-led health program for postoperative patients with early-stage cervical cancer: A randomized controlled trial. Eur J Oncol Nurs 2016;21:174-80.
146. Tian, J. Sexual well-being of cervical cancer survivors under 50 years old and the factors affecting their libido. Gynecol Obstet Invest 2013;76(3):177-81.
147. Fernandes WC, Kimura M. Health related quality of life of women with cervical cancer. Rev Lat Am Enfermagem 2010;18(3):360-7.
148. Wu J, Liu X, Hua K, Hu C, Chen X, Lu X. Effect of nerve-sparing radical hysterectomy on bladder function recovery and quality of life in patients with cervical carcinoma. Int J Gynecol Cancer 2010;20:905-9.
149. Monsky WL, Molloy C, Jin B, Nolan T, Fernando D, Loh S, et al. Quality-of-Life assessment after palliative interventions to manage malignant ureteral obstruction. Cardiovasc Intervent Radiol 2013;36(5):1355-63.
150. Reh AE, Lu L, Weinerman R, Grifo J, Krey L, Noyes N. Treatment outcomes and quality-of-life assessment in a university-based fertility preservation program: Results of a registry of female cancer patients at 2 years. J Assist Reprod Genet 2011;28(7):635-41.
151. Pham A, Yondorf MZ, Parashar B, Scheff RJ, Pannullo SC, Ramakrishna R, et al. Neurocognitive function and quality of life in patients with newly diagnosed brain metastasis after treatment with intra-operative cesium-131 brachytherapy: a prospective trial. J Neurooncol 2016;127(1):63-71.
152. Selman LE, Higginson IJ, Agupio G, Dinat N, Downing J, Gwyther L, et al. The "Spirit 8" successfully captured spiritual well-being in African palliative care: factor and Rasch analysis. J Clin Epidemiol 2012;65(4):434-43.
153. Kamimura A, Myers K, Ashby J, Trinh HN, Nourian MM, Reel JJ. Women in free clinics: An assessment of health-related quality of life for prevention and health education. J Community Health 2015;40(4):793-801.
154. Ben-Arye E, Schiff E, Raz OG, Samuels N, Lavie O. Integrating a complementary medicine consultation for women undergoing chemotherapy. Int J Gynecol Obstet 2014;124(1):51-4.
155. Dent OF, Galt E, Chapuis PH, Yuile P, Sinclair G, Bokey EL. Quality of life in patients undergoing treatment for chronic radiation-induced rectal bleeding. Br J Sur 1998;85(9):1251-4.
156. Karakayali FY, Tezcaner T, Ozcelik U, Moray G. The outcomes of ultralow anterior resection or an abdominoperineal pull-through resection and coloanal anastomosis for radiation-induced recto-vaginal fistula patients. J Gastrointest Surg 2016;20(5):994-1001.
